# Genetic and Molecular Characterization Revealed the Prognosis Efficiency of Histone Acetylation in Pan-Digestive Cancers

**DOI:** 10.1155/2022/3938652

**Published:** 2022-04-05

**Authors:** Tao Zhang, Bofang Wang, Baohong Gu, Fei Su, Lin Xiang, Le Liu, Xuemei Li, Xueyan Wang, Lei Gao, Hao Chen

**Affiliations:** ^1^Department of Oncology, The First Hospital of Lanzhou University, Lanzhou 730000, China; ^2^Lanzhou University Second Hospital, Lanzhou 730000, China; ^3^Cancer Center, Lanzhou University Second Hospital, Lanzhou 730000, China; ^4^Key Laboratory of Digestive System Tumors, Lanzhou University Second Hospital, Lanzhou 730000, China

## Abstract

The imbalance between acetylation and deacetylation of histone proteins, important for epigenetic modifications, is closely associated with various diseases, including cancer. However, knowledge regarding the modification of histones across the different types of digestive cancers is still lacking. The purpose of this research was to analyze the role of histone acetylation and deacetylation in pan-digestive cancers. We systematically characterized the molecular alterations and clinical relevance of 13 histone acetyltransferase (HAT) and 18 histone deacetylase (HDAC) genes in five types of digestive cancers, including esophageal carcinoma, gastric cancer, hepatocellular carcinoma, pancreatic cancer, and colorectal cancer. Recurrent mutations and copy number variation (CNV) were extensively found in acetylation-associated genes across pan-digestive cancers. HDAC9 and KAT6A showed widespread copy number amplification across five pan-digestive cancers, while ESCO2, EP300, and HDAC10 had prevalent copy number deletions. Accordingly, we found that HAT and HDAC genes correlated with multiple cancer hallmark-related pathways, especially the histone modification-related pathway, PRC2 complex pathway. Furthermore, the expression pattern of HAT and HDAC genes stratified patients with clinical benefit in hepatocellular carcinoma and pancreatic cancer. These results indicated that acetylation acts as a key molecular regulation of pan-digestive tumor progression.

## 1. Introduction

Posttranslational modifications (PTMs) are chemical alterations of amino acids that act as regulatory switches that extend the functional execution of proteins and regulate protein interactions in cell signaling networks [1]. Histone function is modulated by PTMs such as ubiquitination and acetylation, and PTMs have been increasingly observed in numerous biological processes [2, 3]. Specific mutations in PTM sites may alter networks and lead to changes in the cellular phenotype that are involved in disease development [4, 5]. Previous studies have shown that abnormalities that emerge during PTMs can both activate carcinogenic pathways and suppress the associated control mechanisms, resulting in alterations to the transcription of oncogenes and tumor suppressor genes [6]. More than 100 types of PTMs have been described to date [7].

As an important form of PTM, protein acetylation is associated with tumor-related diseases. Acetylation is the reversible addition of the N-terminal *ε*-amino group of a lysine to histones by two families of enzymes, histone acetyltransferases (HATs) and histone deacetylases (HDACs). Hence, abnormal alternations in histone acetylation are associated with the silencing of tumor suppressor genes and cancer progression [8, 9]. For example, Fraga et al. found that histone H4 is abnormally altered in human cancer with loss of acetylation at lysine 16 and an additional alteration at lysine 20 [10]. In addition, protein acetylation can affect multiple biological behaviors in eukaryotes, such as transcription regulation, energy metabolism, stress response, cell signal transduction, protein folding and proliferation, and apoptosis [11–14]. Wu et al. reported that HMGA2 acetylation can enhance its binding to the target gene and suppress its ubiquitination and proteasome degradation, resulting in HMGA2 accumulation. This in turn promotes the growth of esophageal squamous cell carcinoma [15]. Previous studies on histone acetylation in tumors have suggested that this reverse process is mainly dependent on the activity of HATs and HDACs. HAT1 is highly expressed in multiple cancers and is associated with poor patient prognosis. As an inducible gene, HAT1 assists in histone production, acetylation, and glucose metabolism, enhancing the proliferation of tumor cells [16].

Digestive cancers were reported the leading cause of cancer-related death worldwide and have high risks of morbidity [17]. Previously, the role of histone methylation in the development of digestive cancers was systematically investigated [18]. As another critical form of posttranslational modifications, protein acetylation was also studied in cancer development, but much effort was usually focused on single cancer. Therefore, this study proposes the possibility of comprehensively investigating the histone acetylation-associated genes in pan-digestive tract cancer.

**Figure 1:**
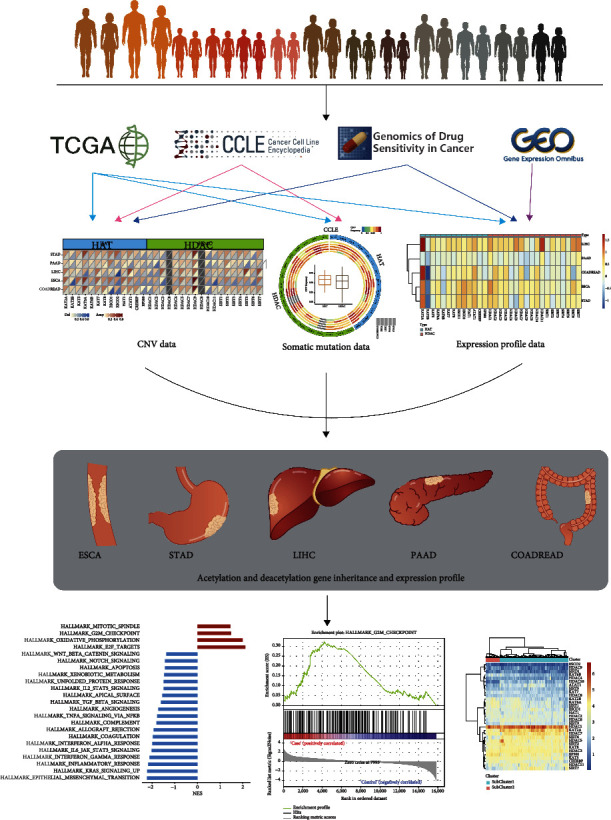
Flow chart of this research. Here, we analyzed HAT and HDAC gene expressions, CNV patterns, and prognostic relevance of ESCA, STAD, LIHC, PAAD, and COADREAD samples from TCGA, GDSC, CCLE, and GEO databases.

The Cancer Genome Atlas (TCGA) database contains the largest number of publicly available pan-digestive cancer samples and serves as a cornerstone for research. To date, 13 classical HATs and 18 HDACs have been documented in the human proteome [19], each of which is involved to varying degrees in the key steps of human physiological and pathological processes. However, the role of the HAT and HDAC gene families in the development of pan-digestive cancers remains unclear. This study was aimed at analyzing the functions and mechanisms of the above genes in pan-digestive cancers to ultimately identify novel treatment targets. Towards this goal, we analyzed the relationship between HAT- and HDAC-regulated gene alterations and explored the clinical prognostic value of HATs and HDACs (Figure 1).

## 2. Materials and Methods

### 2.1. Collection of Multiomics Data of Pan-Digestive Cancer Samples

The publicly available mutation annotation files and CNV information of 5 pan-digestive cancer samples from TCGA were downloaded from the University of California Santa Cruz (UCSC) Xena browser (https://xenabrowser.net/datapages/). The expression data of independent validation datasets for each cancer type were also collected from the ArrayExpress database of EMBL-EBI (https://www.ebi.ac.uk/arrayexpress/) and the Gene Expression Omnibus (GEO) (https://www.ncbi.nlm.nih.gov/geo/) database (Table 1).

To further depict the somatic mutations and CNV features of HAT and HADC genes, cancer cell lines were retrieved and analyzed. Mutation and CNV data were obtained from the Cancer Cell Line Encyclopedia (CCLE) (https://portals.broadinstitute.org/ccle) and the Genomics of Drug Sensitivity in Cancer (GDSC) (https://www.cancerrxgene.org/) databases. In total, 199 cell lines across the five types of cancer were identified from the CCLE and 298 from the GDSC database. Several datasets were downloaded from the GEO and the International Cancer Genome Consortium (ICGC) (https://icgc.org/) databases for the prognostic analysis of HATs and HDACs.

### 2.2. Identification of Differentially Expressed Genes

To identify differential expression patterns of HAT and HADC genes, we downloaded raw counts of RNA-seq data for both tumor and normal samples. Then, the R package limma was used to identify the differentially expressed genes. This identification was performed independently for each digestive cancer type. Genes with log2 | FC | ≥1 and adjusted *p* values < 0.05 were identified as differentially expressed genes. The FDR method was applied to adjust the *p* value.

### 2.3. Somatic Mutation and CNV Analysis

Maftools R package was used for data processing. The mutation frequency in each gene was calculated by dividing the number of samples with mutations in the gene by the total number of cancer samples. Variants that affect protein coding (i.e., nonsynonymous mutations) were retained, including missense, nonsense, translation start site, in-frame, frameshift, splice site, and nonstop mutations. We further analyzed CNV alterations of acetylation regulators using the cBioPortal and applied significant targets in the cancer algorithm (GISTIC 2.0), which was provided by GenePattern (https://cloud.genepattern.org/gp/pages/index.jsf). The parameters in the GISTIC method were set at *Q* ≤ 0.05 for the significance of alternation and at 0.95 for the confidence level of the peak interval.

### 2.4. Correlation Analysis between HAT and HADC Genes and Oncogenic Pathway Activity

We first calculated the activity of cancer hallmark pathways. After scaling the FPKM expression value into a *z*-score statistic using the R package zFPKM, we performed gene set variation analysis (GSVA) to obtain the activity score of each pathway. The Pearson correlation coefficient (PCC) was used to measure the associations between the expression of acetylation and deacetylation genes and the activity score of each pathway. The ∣PCC | >0.5 with FDR-adjusted *p* value < 0.01 was considered the significance threshold.

### 2.5. Gene Set Enrichment Analysis

Gene Set Enrichment Analysis (GSEA) was performed using the GSEA v2.0 tool (http://www.broad.mit.edu/gsea/). Before computation, we converted the mouse gene ID in the expression profile of the KAT6A knockdown model (GSE108242) into human homologous genes using R package homologene.

### 2.6. Survival Analysis of Acetylation and Deacetylation Genes

To inspect the survival correlation of each acetylation and deacetylation gene, we divided the samples based on the mean value of gene expression. Then, the log-rank test was used to determine the differences between high-expression and low-expression groups. In addition, the hazard ratio (HR) and the 95% confidence interval were calculated using the univariate Cox regression. The Kaplan-Meier survival curves with the log-rank test were performed to determine the differences among different patient groups. This process was performed using the survival R package. Statistical significance was set at *p* < 0.05.

## 3. Results

### 3.1. Widespread Genetic Alterations of HAT and HDAC Genes in Pan-Digestive Cancers

**Figure 2:**
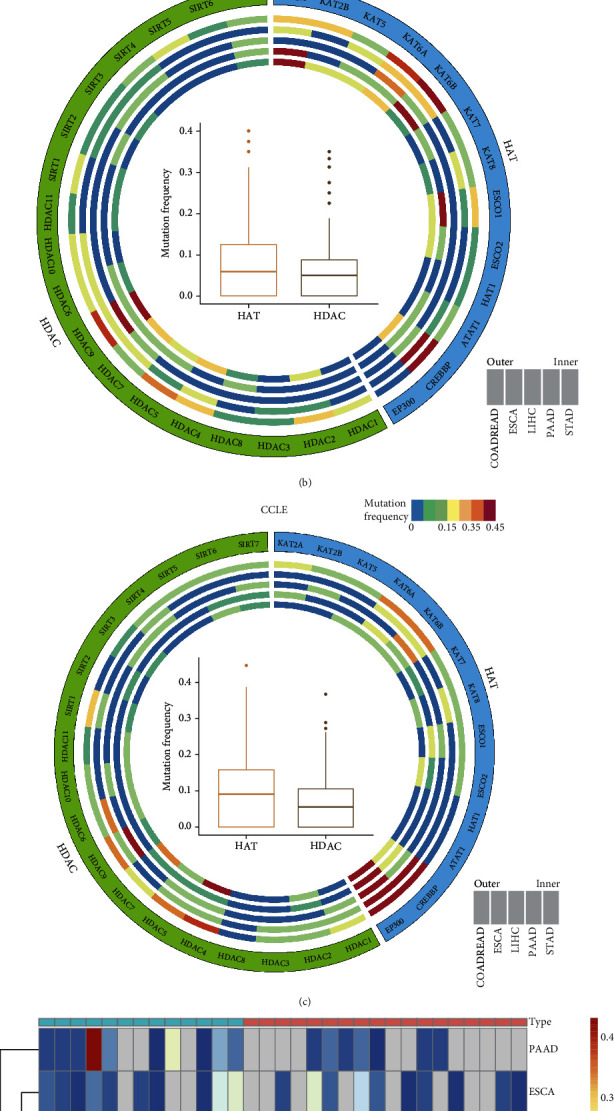
Widespread genetic alterations in HAT and HDAC genes for 5 digestive system cancers. (a) The proportion of HAT and HDAC genes. (b and c) Mutation frequency distributions of HAT and HDAC genes in samples from the GDSC database and CCLE database, respectively. Each circus represents one specific cancer type, and bar graphs within the circle indicated the mutation frequency of each gene. The inner boxplot represented the overall mutation frequency of HAT and HDAC genes in all five digestive cancer types. (d) Scaled mutation frequency of HAT and HDAC genes in 5 pan-digestive system cancers from TCGA.

Previous studies identified 13 acetylation genes (KAT2A, KAT2B, KAT5, KAT6A, KAT6B, KAT7, KAT7, ESCO1, ESCO2, HAT1, ATAT1, CREBBP, and EP300) and 18 deacetylation genes (HDAC1, HDAC2, HDAC3, HDAC4, HDAC5, HDAC6, HDAC7, HDAC8, HDAC9, HDAC10, HDAC11, SIRT1, SIRT2, SIRT3, SIRT4, SIRT5, SIRT6, and SIR7) (Figure 2(a)). Similar analyses of the GDSC and CCLE datasets showed consistent findings with those of TCGA analysis. That is, CREBBP and EP300 had relatively high mutation frequencies in the GDSC and CCLE cell lines (Figures 2(b) and 2(c)). Analysis of the global mutation frequency of the above genes showed that the frequency ranged from 0.014 to 0.474 in the five types of pan-digestive cancers (Figure 2(d)). We found a higher mutation frequency in the acetylated than deacetylated gene group. In addition, mutation of the acetylated and deacetylated gene was likely to be cancer-specific. For example, KAT6A showed a relatively higher mutation frequency in pancreatic and gastric cancers, and the majority of the mutations were missense mutations (Supplementary Figure 1), whereas CREBBP showed the highest mutation frequency than other genes in gastric and colorectal cancers. The difference is that CREBBP enriched different types of mutations (Supplementary Figure 1).

### 3.2. The Landscape of CNV Alterations of HATs and HDACs in Pan-Digestive Cancers

**Figure 3:**
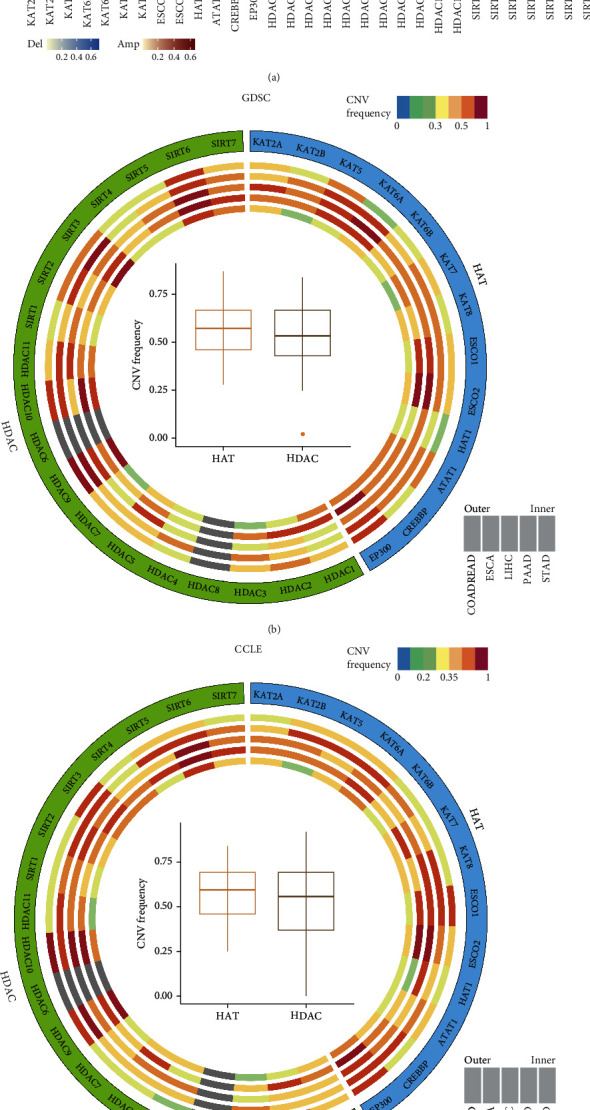
CNV alteration landscape of HAT and HDAC genes in 5 digestive system cancers. (a) Frequency of copy number amplification and deletion of genes across cancer types from TCGA. The gray part indicated no CNV alterations of the genes in the corresponding cancer type. (b and c) CNV frequency of HAT and HDAC genes in samples from the GDSC database and CCLE database, respectively. Each circus represents one specific cancer type, and bar graphs within the circle indicated the mutation frequency of each gene. The inner boxplot represented the overall mutation frequency of HAT and HDAC genes in all five digestive cancer types.

The analysis of the CNV alteration features for all acetylation and deacetylation genes in TCGA, GDSC, and CCLE databases showed a high prevalence of CNV alterations (Figure 3). In the TCGA dataset, HDAC9 and KAT6A showed widespread CN amplification across all five types of pan-digestive cancers (Figure 3(a)). Similar conclusions were obtained in the GDSC and CCLE datasets (Figures 3(b) and 3(c)). Moreover, ESCO2, EP300, and HDAC10 had prevalent CN deletions in the TCGA dataset. We also analyzed alternations in the different CNV expression patterns of these genes and found that the CN amplification group had higher expression levels than the CN deletion group (Supplementary Figure 2), and this was partially verified in the cell line dataset. Although there was no significant difference in CNVs between acetylated and deacetylated genes, acetylation and deacetylation undoubtedly played important roles in pan-digestive cancers.

### 3.3. Differential Expressions of HAT and HDAC Genes

**Figure 4:**
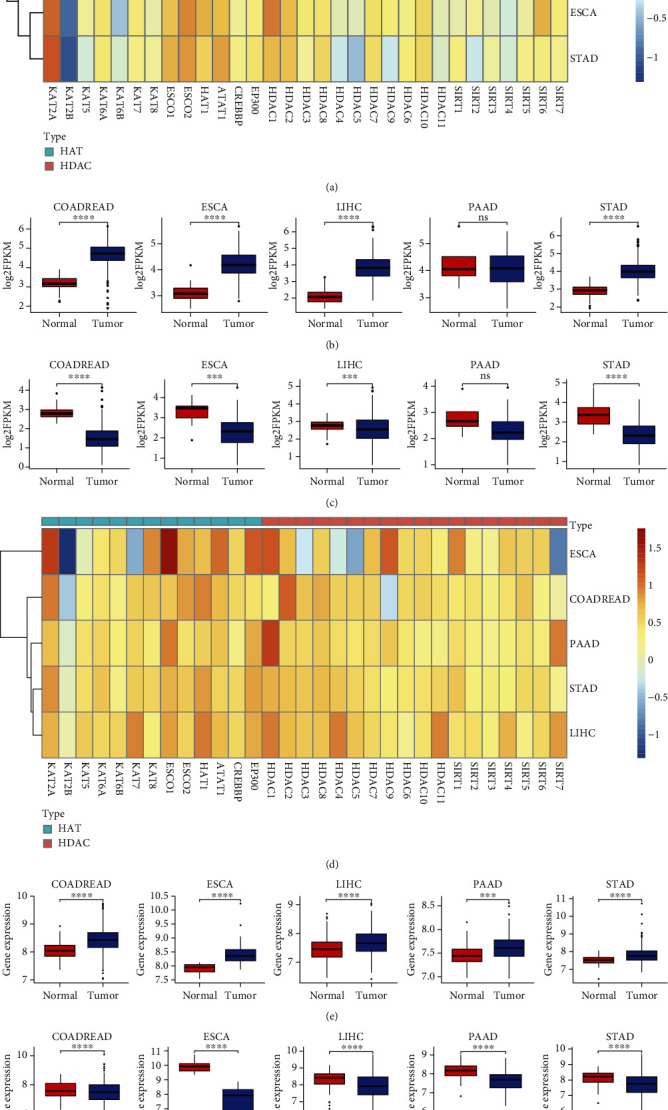
Differential expression analysis of HAT and HDAC genes in 5 pan-digestive system cancers. (a) The heat map indicated the fold changes of HAT and HDAC genes (column) in 5 pan-digestive system cancers (row) compared to adjacent normal samples from TCGA, with red representing upregulated genes and blue representing downregulated genes in tumor samples. (b and c) Comparison of expression levels between tumor and normal samples for KAT2A and KAT2B. Significance was assessed by the Wilcoxon rank-sum test. (d) The same as panel (a) but for independent validation sets (Table 1). (e and f) The same as panels (b and c) but for independent validation sets. ns: *p* > 0.05, ^∗^*p* < 0.05, ^∗∗^*p* < 0.01, and ^∗∗∗^*p* < 0.0001.

We further analyzed the differential expression of these genes between cancer and adjacent normal samples of five pan-digestive cancer types (Figure 4). The results showed that KAT2A had accordingly higher expression, whereas KAT2B had accordingly lower expression in the tumor samples compared to normal samples of five pan-digestive cancers from TCGA (Figures 4(a)–4(c)). The differential expression pattern was in agreement with the copy number status of the two genes; that is, copy number amplification of KAT2A and copy number deletion of KAT2B were found in pan-digestive cancers (Figure 3(a)). Independent validation datasets also supported similar expression patterns of the two genes (Table 1 and Figures 4(d)–4(f)). Notably, the differential expression pattern was in agreement with the copy number status of the two genes; that is, copy number amplification of KAT2A and copy number deletion of KAT2B were found in pan-digestive cancers (Figure 3(a)). We also verified the effect of CNV on expression patterns of these genes and found that the copy number amplification induced higher expression levels than copy number deletion (Supplementary Figure 3).

### 3.4. Oncogenic Pathways Regulated by HAT and HDAC Genes

**Figure 5:**
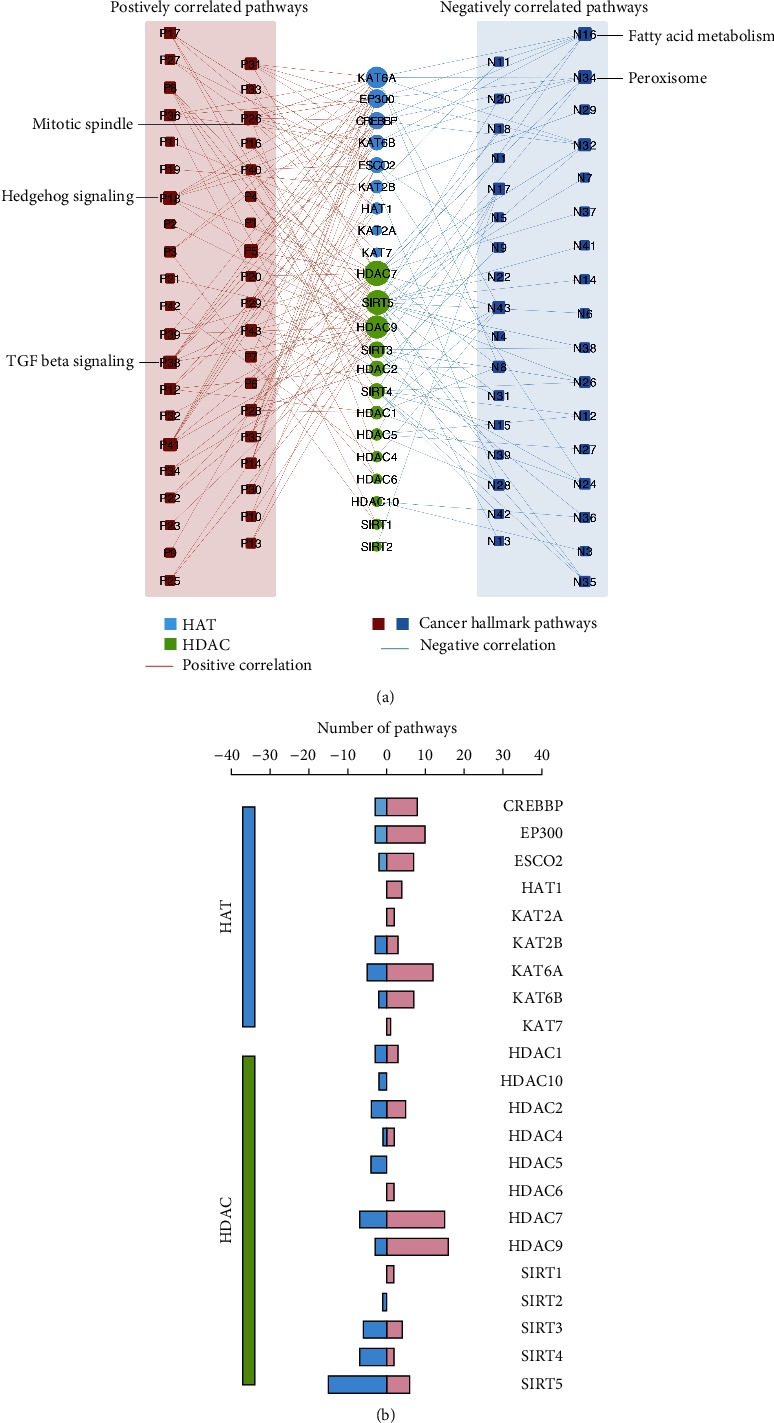
Oncogenic pathways regulated by the HAT and HDAC genes in pan-digestive system cancers. (a) Network diagram demonstrating the correlation between HAT and HDAC genes and cancer pathways. Red represents a positive correlation, and blue represents a negative correlation. The size of the nodes corresponds to the number of links. HDAC group is marked as green color and HAT group is marked as blue color. (b) The number of pathways is correlated with individual HAT and HDAC genes. The right panel is for positively correlated pathways and marked in the pink bar, and the left panel is for negatively correlated pathways and marked in the blue bar. Also, genes are clustered in the blue and green groups which represent the HAT group and HDAC group, respectively.

To further investigate the molecular mechanisms involved in the acetylation and deacetylation of genes in pan-digestive cancers, we examined the correlation between the expression of individual acetylated and deacetylated genes, which were involved in the 50 cancer hallmark-related pathways. We found that the expressions of acetylated and deacetylated genes were correlated with the activation or inhibition of multiple oncogenic pathways (Figure 5). The expressions of KAT6A, HDAC9, HDAC7, and SIRT5 were positively correlated with multiple pathways, including HALLMARK_TGF_BETA_SIGNALING and HALLMARK_HALLMARK_HEDGEHOG_SIGNALING (Figure 5(a)). TGF_BETA_SIGNALING belongs to transforming growth factor *β* (TGF-*β*), which promotes epithelial-mesenchymal transition in late-stage cancer by being highly involved in cell migration [20]. The latter pathway controls cell fate, proliferation, and differentiation [21, 22]. Abnormal activation of these pathways causes tumorigenesis. In addition, different HAT and HDAC genes were associated with distinct cancer pathway alterations. These genes also showed heterogeneous effects of histone modifications (Figure 5(b)).

**Figure 6:**
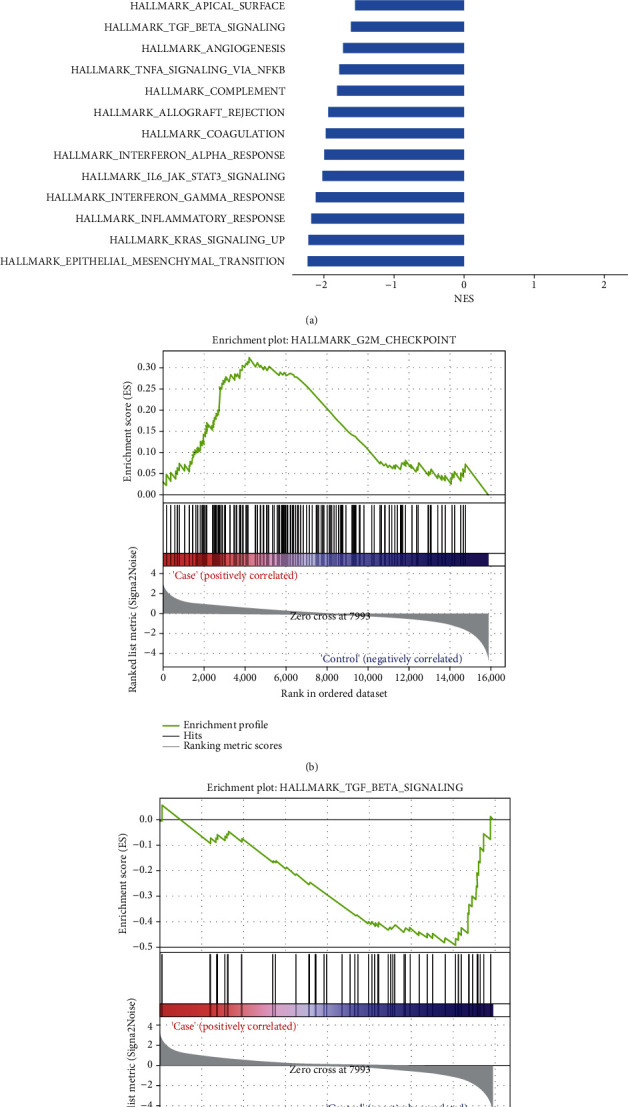
GSEA analysis of KAT6A knockdown model samples. (a) Enriched pathways after KAT6A knockdown. (b and c) Enrichment plot for G2M signal checkpoint pathway and TGF-*β* signaling pathway.

The analysis of the expression profile (GSE108242) obtained from the KAT6A gene knockdown model revealed that the expression of this gene was closely associated with several cancer hallmark-related pathways (Figure 6(a)). G2M signal checkpoint pathway (HALLMARK_G2M_CHECKPOINT) was found upregulated after the knockdown of KAT6A (Figure 6(b)), whereas the TGF-*β* signaling pathway was inhibited after KAT6A knockdown (Figure 6(c)); this observation was also supported by previous analysis [23].

**Figure 7:**
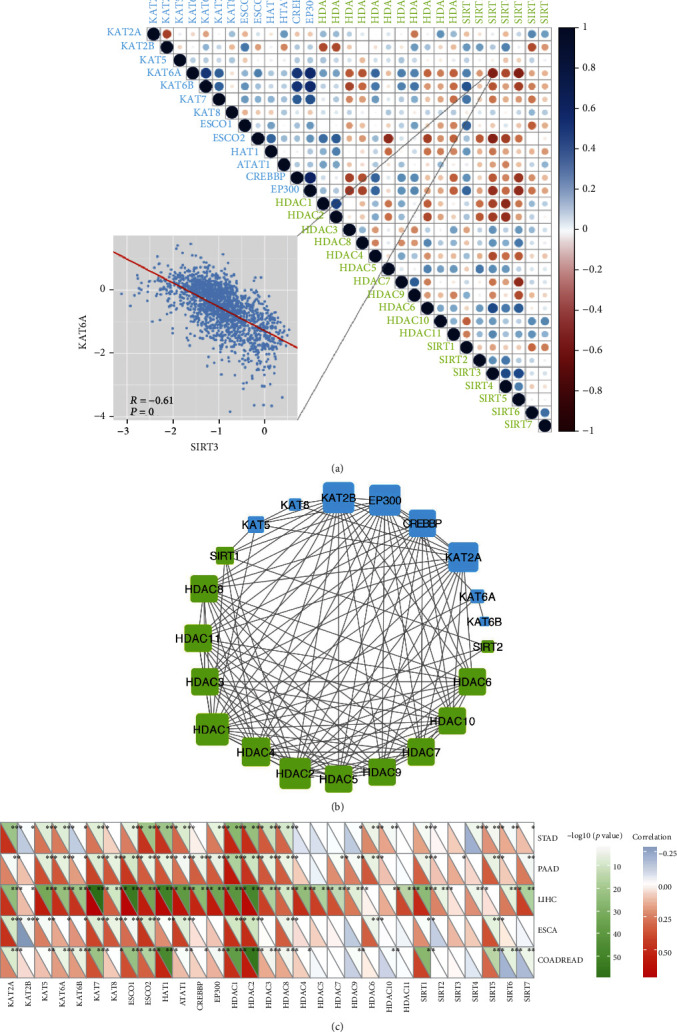
Gene expression relations and PPI network among HAT and HDAC genes. (a) Correlation among the expression of HAT and HDAC genes. The scatter plot shows the correlation between KAT6A and SIRT3. (b) The protein-protein interactions among HAT and HDAC genes. (c) HAT and HVAC genes are associated with PRC2 complex activity in five pan-digestive system cancers. The lower triangle represents the correlation between HAT and HDAC genes and the PRC2 complex. The red color represents the positive correlation and the blue color represents the negative correlation; the upper triangle represents the correlation between HAT and HDAC genes and PRC2 complex; the dark green represents significant correlations.

Genes do not function in isolation, and previous studies have shown the collaboration between HAT and HDAC genes in the context of cancer [1, 24]. Thus, we investigated the coexpression of the acetylated and deacetylated gene groups (Figure 7(a)). We found that HAT and HDAC genes within the same functional class showed highly correlated expression patterns. For instance, the acetylated gene CREBBP was significantly correlated with EP300 (*r* = 0.8043, *p* = 0). There were also negative correlations between HAT and HDAC modifications, such as those in KAT6A and SIRT3 (Figure 7(a), *r* = −0.6087 and *p* = 0). Furthermore, we constructed protein-protein interaction networks for HATs and HDACs and found that they were closely correlated (Figure 7(b)).

### 3.5. Correlation between HAT and HDAC Genes and PRC2 Complex Activity

H3K27me3 is a very important histone modification that plays an important role in X chromosome inactivation, embryonic development, and disease progression by inhibiting gene expression [25–27]. The formation of H3K27me3 is mediated by the polycomb inhibitor complex 2 (PRC2) [28, 29]. We investigated the correlation between the expression of acetylated and deacetylated genes and the activity of the PRC2 complex pathway (BIOCARTA_PRC2_PATHWAY). The results showed that acetylation was positively correlated with PRC2 pathway activity, whereas deacetylation was mostly negatively correlated (Figure 7(c)). The other study showed that the dual suppression of EZH2 and HDAC led to the dissociation of PRC2, and HDAC 1/2 was positively correlated with PRC2 activity [30], which was also demonstrated by our study.

### 3.6. Survival Analysis of HAT and HDAC Family Genes

**Figure 8:**
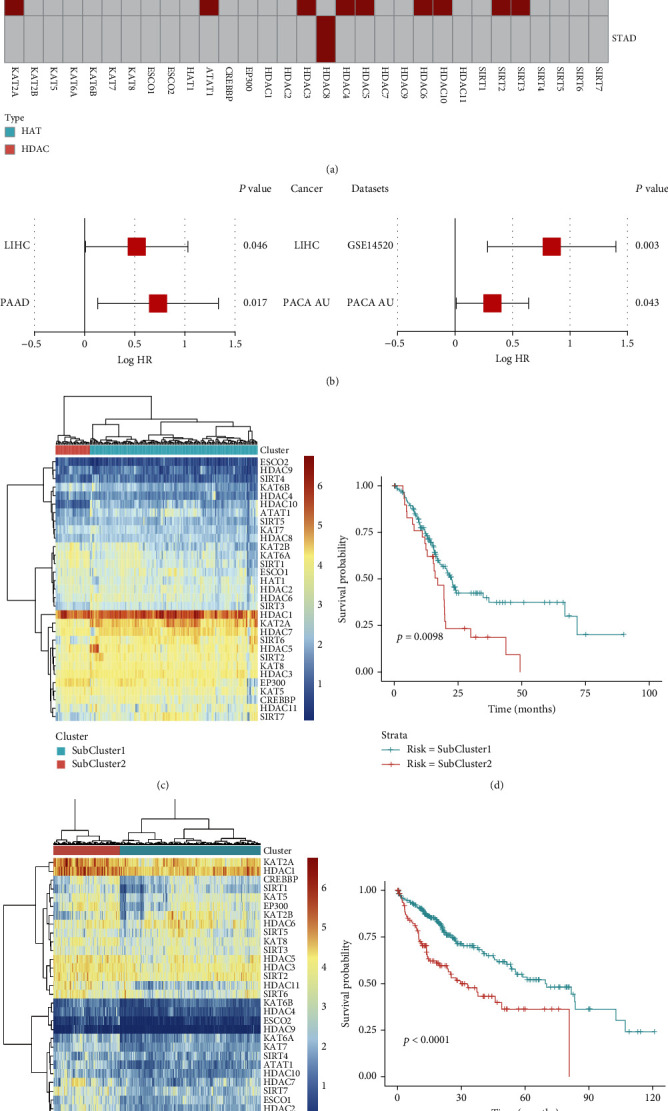
Survival analysis of HAT and HDAC family genes in pan-digestive system cancers. (a) A summary of the correlation between expression of HAT and HDAC genes and patient survival. Red represents a higher expression of HAT and HDAC genes associated with worse survival, and blue represents an association with better survival. (b) The HDAC6 forest map in hepatocellular carcinoma and pancreatic cancer of GEO and ICGC datasets. (c) Heat map showing the clustering for pancreatic cancer patients based on the expression of HAT and HDAC genes. (d) Kaplan-Meier survival plot of patients grouped by the global expression pattern of HAT and HDAC genes. (e) Heat map showing the clustering for hepatocellular cancer patients based on the expression of HAT and HDAC genes. (f) Kaplan-Meier survival plot of patients grouped by the global expression pattern of HAT and HDAC genes.

We further analyzed the correlation between the expression levels of HAT and HDAC genes and the prognosis across five pan-digestive cancers. All HAT and HDAC genes were associated with the overall survival of patients in at least one cancer. The prognoses of gastric cancer, hepatocellular carcinoma, pancreatic cancer, and colorectal cancer were associated with HAT and HDAC genes (Figure 8(a)). We found that 12 HAT and HDAC genes were highly expressed, and the upregulation of these genes was associated with a better prognosis of hepatocellular carcinoma. Nine genes, including KAT2A, were highly expressed in pancreatic cancer and were associated with poor prognosis. Particularly, KAT2A was significantly upregulated in all five types of cancer (*p* = 0.028). In addition, high expression of HDAC6 was associated with a poor prognosis of hepatocellular carcinoma (*p* = 0.046) and pancreatic cancer (*p* = 0.028). The prognostic role of HDAC6 was further verified in the hepatocellular carcinoma (GSE14520) and pancreatic cancer (PACA-AU) datasets in GEO and ICGC (Figure 8(b)), and the findings were consistent with that in the TCGA dataset.

Previous studies indicated that the HAT and HDAC families were associated with the prognosis of hepatocellular carcinoma and pancreatic cancer. Thus, we clustered hepatocellular carcinoma and pancreatic cancer samples based on the integration of HAT and HDAC gene expressions (Figures 8(c) and 8(e)) and compared the survival differences between the clusters. The two clusters of patients showed significant differences in overall survival (log-rank test *p* = 0.0098 for pancreatic cancer, Figure 8(d); log-rank test *p* < 0.0001 for hepatocellular carcinoma, Figure 8(f)). Finally, a graphic prognostic nomogram based on the sample clusters was developed for 0.5-, 1-, 3-, and 5-year prediction of OS. The tumor stage, age, and gender were also included (Supplementary Figure 4). As a whole, the expression of histone acetylation genes might help guide the prognostic status of patients.

## 4. Discussion

In this study, we comprehensively analyzed multiomics data on acetylation-associated signatures. According to the previous analysis, metabolomics, transcriptomics, epigenetics, and proteomics data were integrated to investigate the role of the acetylation-related signatures in cancer. Multiomics data enable us to predict novel functional interactions between molecular mediators at multiple levels [31, 32]. Additionally, these data can be potentially used to uncover crucial biological observations into hallmark pathways that would otherwise not be determined through single-omics studies [33]. PTM acetylation has recently received widespread attention. Zheng et al. found that HDAC3-mediated deacetylation induces ENO2 activation and glycolysis enhancement, thus promoting the metastasis of pancreatic cancer [34]. Pote et al. reported that HAT hMOF promotes vascular invasion in hepatocellular carcinoma [35]. KAT2A was highly expressed in pancreatic cancer; its mediated expression of 14-3-3*ζ* and *β*-catenin has been shown to promote glycolysis, proliferation, epithelial-mesenchymal transition, and migration in pancreatic cancer cells [36]. However, molecular descriptions of the 13 HAT and 18 HDAC gene signatures in pan-digestive cancer tissues and cell lines are still lacking. As such, we performed a comprehensive analysis of acetylation signatures to provide a useful resource for future related research.

CNVs are nearly ubiquitous in cancer and markedly affect the cancer genome as a key type of genomic variation [37–39]. The CNV landscape of genes varies across different types of cancer, and specific CNVs are related to cancer outcomes [40]. Some genes with CN amplification, such as KAT2A, are highly expressed. Yin et al. reported upregulated KAT2A mRNA levels, induced by the transcription factors c-MYC and E2F1, in colon cancer [41]. Some genes with CN deletions, such as KAT2B, have low expression. Particularly, KAT2B was downregulated in all five cancers as well as in approximately 3000 other cancer samples. These findings are consistent with those reported by Li et al. and Ying et al. They showed that KAT2B expression was lower in gastric cancer and hepatocellular carcinoma tissues than that in adjacent normal tissues [42, 43]. Zhang et al. found that ferroptosis is an outcome of metabolic disorders and is closely linked to hepatocellular carcinoma. Importantly, the occurrence of ferroptosis was possibly dependent on the HAT KAT2B [44]. The Kaplan-Meier analysis by Li et al. showed that the downregulation of KAT2B expression was associated with lower overall survival in hepatocellular carcinoma [42]. In our analysis, the CN gains of ESCO2 and HDAC10 were found to be risk factors for colorectal and pancreatic cancer, respectively. Therefore, CNVs of HAT and HDAC genes may be used to predict the prognosis in some cancers.

We evaluated the correlation between the 13 HAT gene signatures and the expression of 18 HDAC genes and found that genes within the same functional class showed significant cooccurrences of genetic alterations and highly correlated expression patterns. However, there was also a high correlation between HAT and HDAC modifications. For instance, the acetylation gene CREBBP was significantly correlated with EP300 expression. EP300, also known as p300 and CREBBP, serves as a scaffold that bridges sequence-specific DNA binding factors and the basal transcriptional machinery. Further, it facilitates transcription through acetylation of histones, transcription factors, and autoacetylation [45–47]. Many studies have shown that EP300 and CREBBP play a major role in promoting cell growth and cell cycle progression [46]. There are more than 16,000 genes in human cells that bind to EP300 and CREBBP in the transcriptional regulation process [48, 49]. In response to DNA damage, EP300 and CREBBP augment the p53-dependent transcriptional activation of genes required for cell cycle arrest and DNA repair [50]. However, to the best of our knowledge, no study investigated the correlation between the expressions of KAT6A and SIRT3 in cancer, despite their potential importance in tumorigenesis.

Prognosis prediction is crucial in cancer management because it can aid in the subsequent clinical treatment of patients. However, although remarkable improvement has been achieved in cancer research over the past several decades, prognostic prediction remains a challenge [39]. Thus, we also attempted to evaluate the impact of HAT gene signatures on cancer prognosis. The univariate Cox regression analysis revealed that over half of the examined genes were associated with prognosis in at least one of the cancers. High KAT2A expression was associated with a poorer prognosis in pancreatic cancer. A risk score based on the expression of HATs and HDACs could be useful for distinguishing between high- and low-risk patients with hepatocellular carcinoma and predicting their prognosis.

There are some limitations to this study. It is difficult to process and analyze several data sources under a unifying framework, given the diversity of methodological work in this area [51]. Genomic mutations and gene expression need to be validated. In addition, some data were collected from retrospective studies wherein certain critical parameters were not recorded, and influencing factors could bias the selection of controls. Further prospective clinical studies are required to validate our findings.

In conclusion, we demonstrated the prevalent genetic and expression alterations of histone modifications across five pan-digestive cancers. These HAT and HDAC gene mutations are highly correlated with the activation and inhibition of cancer pathways and with the prognosis of pancreatic cancer and hepatocellular carcinoma. This systematic analysis of the landscape of molecular alterations and clinical relevance of acetylation lays a critical foundation for understanding the dysregulation of PTM in pan-digestive cancers. Further, it provides insights that can be useful for the development of related therapeutic targets.

## Figures and Tables

**Table 1:** Validation RNA-seq datasets of pan-digestive cancers.

Cancers	No. of normal	No. of tumor	References
Colon/rectum adenocarcinoma	121	1393	E-MTAB-6698
Stomach adenocarcinoma	46	691	E-MTAB-6693
Liver hepatocellular carcinoma	137	264	E-MTAB-6695
Pancreatic adenocarcinoma	70	108	E-MTAB-6690
Esophageal carcinoma	19	21	GSE26886

## Data Availability

The data used to support the findings of this study are included within the article.

## Supplementary Materials

Supplementary Materials Supplementary Figure 1: the summary of mutations in histone acetylation-associated genes of each digestive cancer type from TCGA. Supplementary Figure 2: HDA9, KAT6A, ESCO2, EP300, and HDAC10 expressions in TCGA five pan-digestive system cancers. Supplementary Figure 3: KAT2A and KAT2B expressions in TCGA five pan-digestive system cancers. Supplementary Figure 4: the constructed nomogram for prognosis in pancreatic cancer and hepatocellular carcinoma patients from TCGA.
