# Supplementary material for: Genetic and Molecular Characterization Revealed the Prognosis Efficiency of Histone Acetylation in Pan-Digestive Cancers
Source: J Oncol. 2022 Apr 5;2022:3938652. doi: 10.1155/2022/3938652 (PMC9005301; doi:10.1155/2022/3938652)
Supplement: Supplementary Materials — Supplementary Figure 1: the summary of mutations in histone acetylation-associated genes of each digestive cancer type from TCGA. Supplementary Figure 2: HDA9, KAT6A, ESCO2, EP300, and HDAC10 expressions in TCGA five pan-digestive system cancers. Supplementary Figure 3: KAT2A and KAT2B expressions in TCGA five pan-digestive system cancers. Supplementary Figure 4: the constructed nomogram for prognosis in pancreatic cancer and hepatocellular carcinoma patients from TCGA. [file 3938652.f1.docx]

## Supplementary Materials


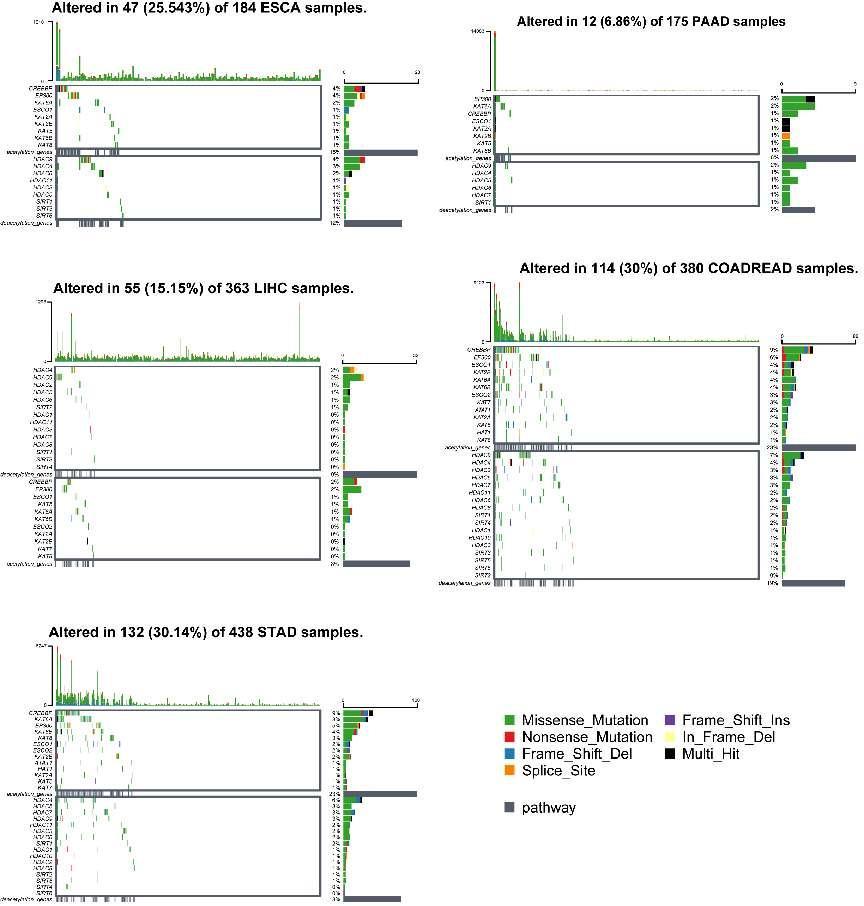


**Supplementary Figure 1.** The summary of mutations in histone acetylation associated genes of each digestive cancer type from TCGA.


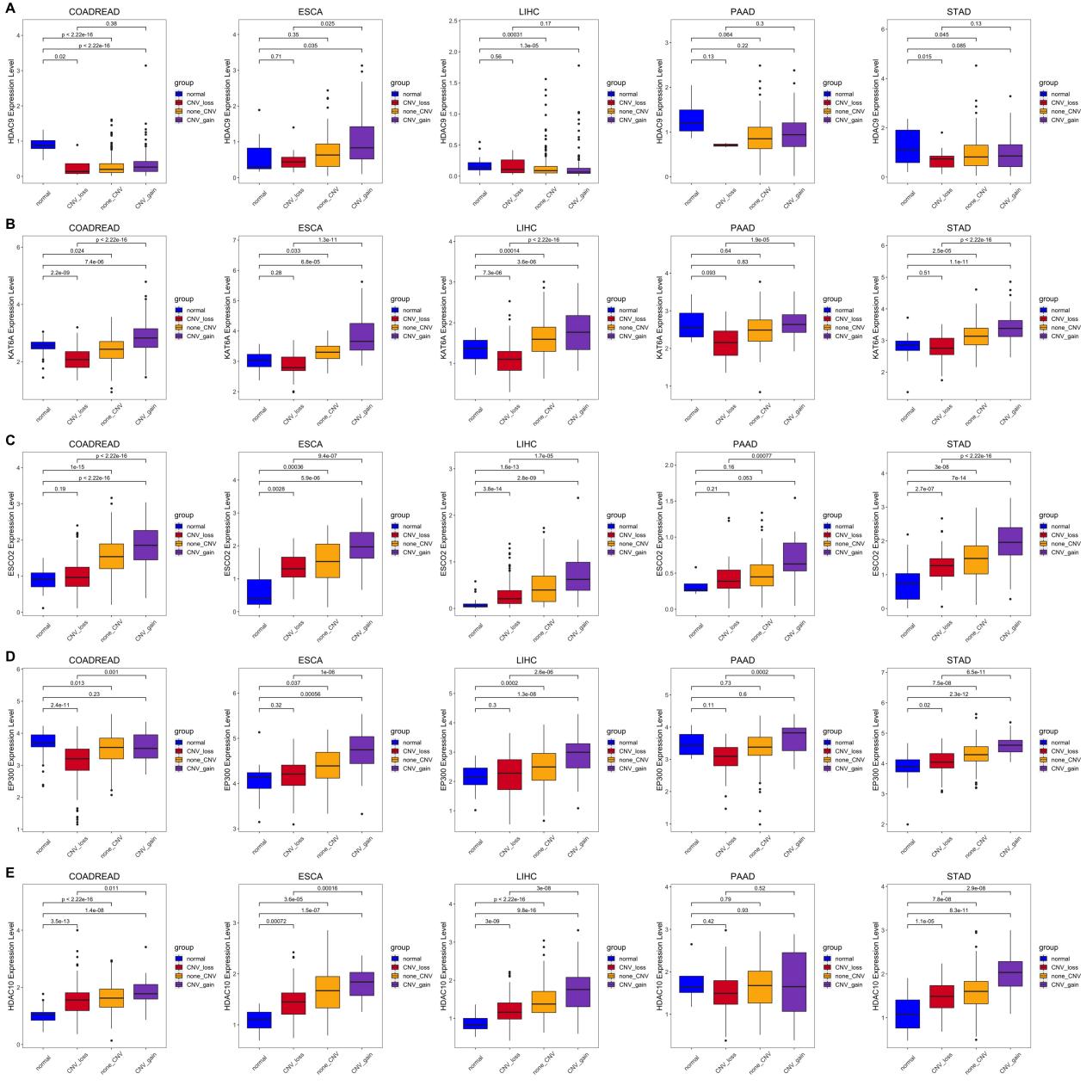


**Supplementary Figure 2**. HDA9, KAT6A, ESCO2, EP300, HDAC10 expression in TCGA five pan-digestive system cancers. The blue box is for normal tissues, red box is for CNV_loss in tumor tissues, the yellow box is for wild type in tumor tissues and the purple box is for CNV_gain in tumor samples. A, HDAC9; B, KAT6A; C, ESCO2; D, EP300 and E, HDAC10.


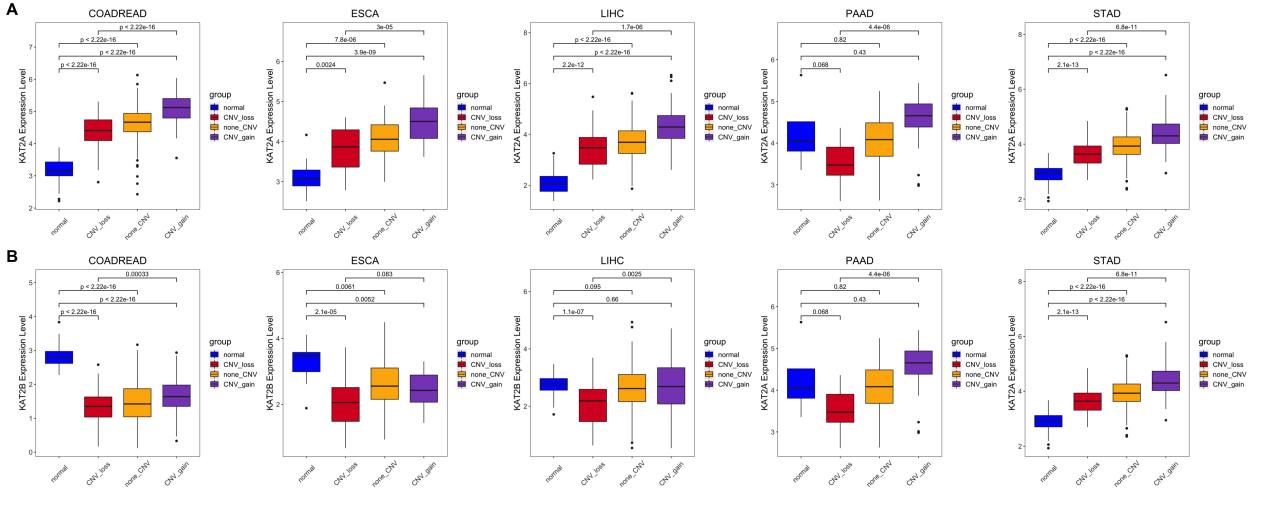


**Supplementary Figure 3**. KAT2A and KAT2B expression in TCGA five pan-digestive system cancers. The expression of “writers” among CNV groups in five pan-digestive system cancers. A. KAT2A gene expression in the five TCGA cancer types. The blue box is for normal tissues, red box is for CNV_loss in tumor tissues, the yellow box is for none_CNV in tumor tissues and the purple box is for CNV_gain in tumor samples. B. KAT2B, gene expression in the five TCGA cancer types. The blue box is for normal tissues, red box is for CNV_loss in tumor tissues, the yellow box is for none_CNV in tumor tissues and the purple box is for CNV_gain in tumor samples. ns: p > 0.05, *: p < -0.05, **: p < -0.01, ***: p < -0.0001, The sample size for each group based on the CNV alteration (CFI, CNV_loss/ CNV_gain/ normal/ none_CNV = 138/18/54/305)


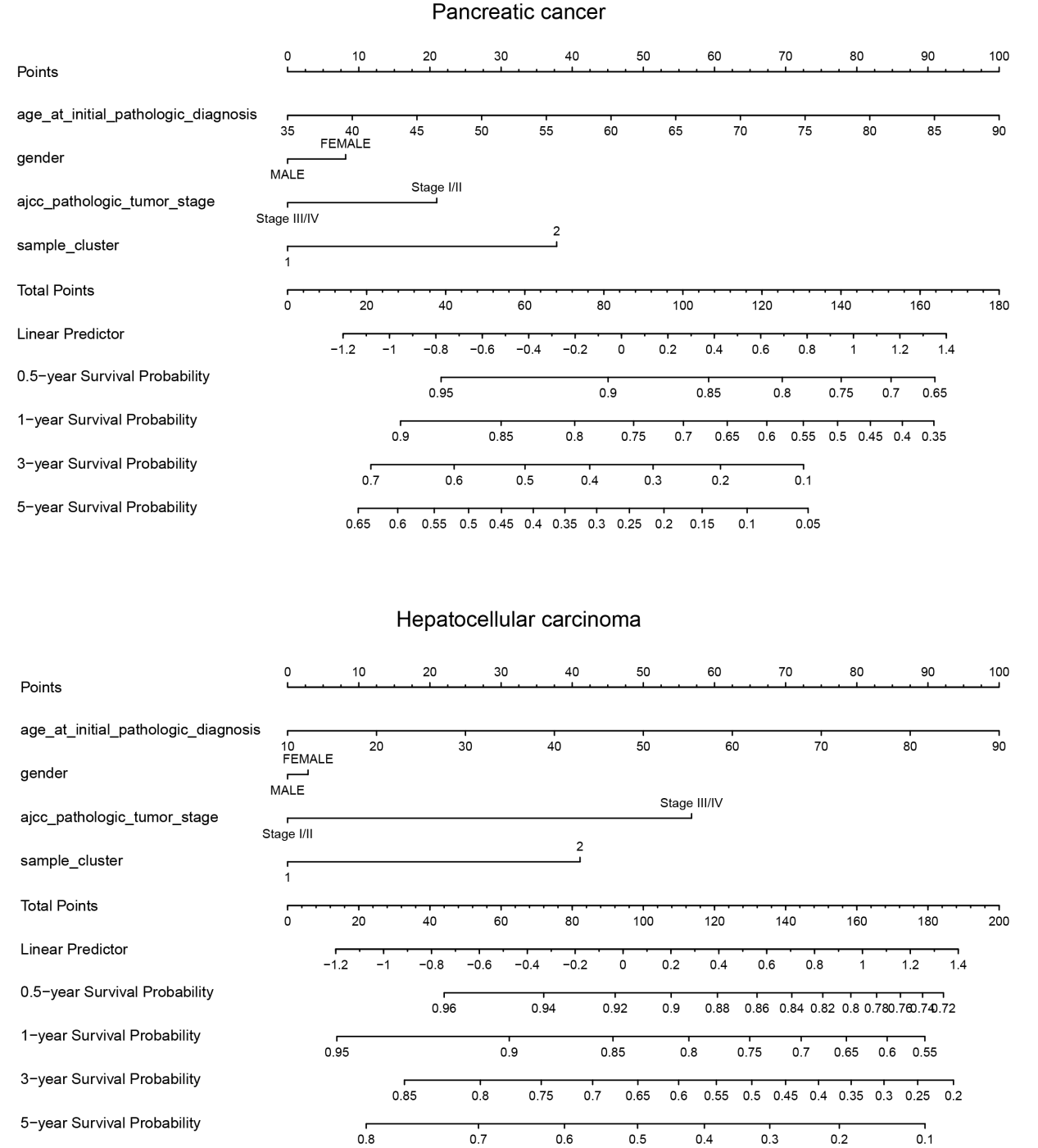


**Supplementary Figure 4**. The constructed nomogram for prognosis in pancreatic cancer and hepatocellular carcinoma patients from TCGA.
